# CK5/6 Expression in Molecular Subtypes of Invasive Ductal Carcinoma

**DOI:** 10.7759/cureus.72608

**Published:** 2024-10-29

**Authors:** Rafeya Yasin, Ghazi Zafar, Fatima Rooman Ali Syed, Sameen Afzal, Maryam Fatima, Zonaira Rathore, Akhtar Chughtai, Anila Chughtai

**Affiliations:** 1 Histopathology, Chughtai Institute of Pathology, Lahore, PAK

**Keywords:** ck5/6, her-2-enriched, luminal a, luminal b, triple-negative breast cancer, triple-negative breast carcinoma

## Abstract

Background

Breast cancer (BC) is the leading cause of cancer-related deaths in women worldwide. There has been a significant increase in the incidence of BC in Pakistan. Family history, older age, obesity, tobacco use, oral contraceptive use, early menarche, and hormonal replacement therapy are among the major risk factors. The most common histological subtype of BC is invasive ductal carcinoma (IDC). Molecular subtypes of BC include mainly Luminal A, Luminal B, human epidermal growth factor receptor 2 (HER-2) enriched, and triple-negative BC subtypes, with the triple-negative subtype having the worst prognosis. CK5/6 serves as a basal keratin biomarker. This aimed to assess the expression of CK5/6 in IDC of the breast belonging to different molecular classes and to compare its expression with traditionally defined prognostic factors for different molecular subtypes.

Methodology

A cross-sectional, observational study was conducted at the Chughtai Institute of Pathology after approval from the Institutional Review Board (approval number: 1198/IRB/CIP). All cases during a period of six months (April 2023 to September 2023) were sampled using non-probability convenient sampling. All mastectomy samples diagnosed as IDC were included in the study. After standard tissue processing, paraffin tissue blocks and slides were prepared followed by hematoxylin & eosin staining. Hormonal receptors (estrogen receptor, progesterone receptor, HER-2) were assessed for cases to segregate them into molecular subtypes. CK5/6 antibody was then applied and the data were collected on a pre-designed proforma. SPSS version 25.0 (IBM Corp., Armonk, NY, USA) was used for data analysis.

Results

Of a total of 85 cases, 19 (22.3%) were positive for CK5/6. Of these 19 cases, the majority (68%, p = 0.001) belonged to the triple-negative class of tumors, comprising 13 cases. No case from the Luminal A class showed expression for CK5/6 stain (p = 0.028). Overall, four cases of the Luminal B subtype showed CK5/6 positivity (10.8%, p = 0.022) while two cases of the HER-2-enriched subtype were positive for the stain (33.3%, p > 0.05). These results were analyzed in relation to different prognostic factors. The majority of CK5/6-positive cases showed lymphovascular invasion (42%) and belonged to grade 3 tumors (57.8%).

Conclusions

The expression of CK5/6 in IDC of the breast is associated with poor prognostic factors such as triple-negative molecular subtypes, high histological grade, lymphovascular invasion, positive nodal status, and high pathological stage.

## Introduction

Breast cancer (BC) is the most commonly diagnosed cancer and the leading cause of cancer-related deaths in women worldwide [1,2]. In 2023, the cancer statistics report showed 300,590 new cases of BC and 43,700 deaths related to BC in the United States [3]. The overall incidence of BC is comparatively lower in Asia than in Western countries; however, there has been a drastic increase in the incidence of BC in Pakistan with an estimate of one in nine women having a lifetime risk of developing BC [4]. Family history, older age, obesity, tobacco use, oral contraceptive use, early menarche, and hormonal replacement therapy are among the major risk factors for the development of BC [2]. It is a highly heterogeneous disease with great variability in the clinical treatment and prognosis among different patients [5]. The most common histological subtype of BC is invasive ductal carcinoma (IDC), also referred to as invasive breast carcinoma of no special type (IBC NST) [6,7].

The treatment strategies for IBC NST include a surgical approach, radiotherapy, chemotherapy, hormonal therapy, and immune therapy which largely depend on the expression of hormone receptors in the tumor cells, including estrogen (ER), progesterone (PR), and human epidermal growth factor (HER-2) receptors [8]. Based on hormone receptors, IBC NST is divided into four distinct molecular subtypes, including Luminal A, Luminal B, and HER-2-enriched, and triple-negative class (TNC) [9]. Luminal A tumors have the best prognosis while TNC has the worst prognosis [10].

CK5/6 is a basal keratin biomarker whose expression in BC has been linked with aggressive behavior and has traditionally been reported in IDC belonging to TNC. It has been linked to worse prognostic characteristics such as high histological grade, larger tumor size, and decreased disease-free survival [11-14]. This study aimed to assess the expression of CK5/6 in IDC belonging to different molecular classes and to establish a correlation with traditionally defined prognostic factors.

## Materials and methods

A cross-sectional, prospective study was conducted at the Chughtai Institute of Pathology after obtaining approval from the institutional review board (approval number: 1198/IRB/CIP). This study was conducted over a six-month period, i.e., from April 2023 to September 2023. Sampling was done using non-probability convenient sampling, and all cases booked during the above-mentioned study period were included in the study. A total of 733 cases were booked in this period, and 85 cases were included which fulfilled the study criteria. All mastectomy specimens diagnosed as IDC were included in the study. All other types of BCs, incisional biopsies, and poorly preserved samples with autolyzed morphologies were excluded from the study.

After standard processing of tissue samples, paraffin tissue blocks were made and slides were prepared followed by hematoxylin & eosin staining. Hormonal receptors (ER, PR, HER-2) were assessed for all cases using the Autostainer Link 48 provided by DAKO. The data were then grouped into molecular classes based on hormone receptor expression as per Allred scoring. CK5/6 antibody (Monoclonal Mouse Anti-human Cytokeratin 5/6 Clone D5/16 B4 from DAKO) was applied to all cases using the same protocol. Membranous staining of more than 10% of tumor cells with CK5/6 antibody was considered positive. Variables such as age and gender, histological grade (G), pathological tumor stage (pT), regional lymph node status, lymphovascular invasion (LVI), and perineural invasion (PNI) were noted. All data were collected on a pre-designed proforma and the results were analyzed using SPSS version 25.0 (IBM Corp., Armonk, NY, USA).

## Results

**Figure 1:**
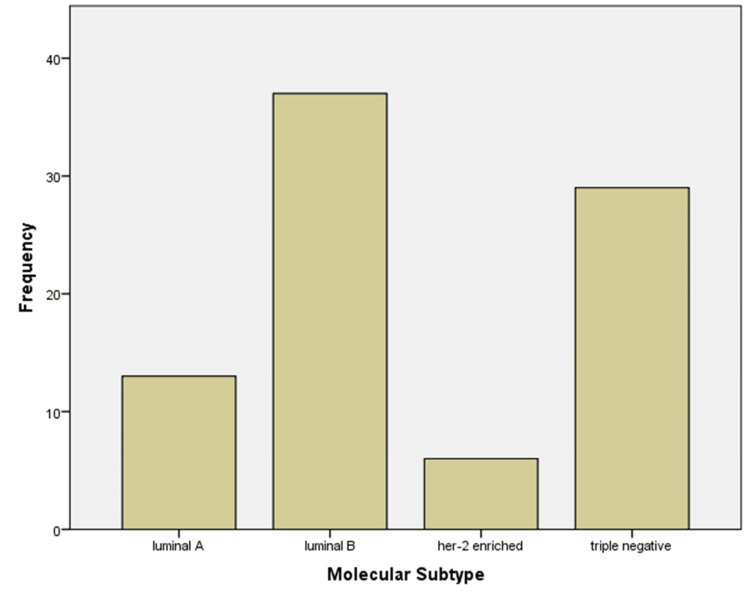
Frequency of molecular subtypes of invasive ductal carcinoma in the study population.

A total of 85 cases of IDC were included in the study. The most common molecular subtype was Luminal B (n = 37, 43.53%), followed by TNC (n = 29, 34.12%), Luminal A (n = 13, 15.29%), and HER-2-enriched (n = 6, 7.06%) (Figure 1).

Of a total of 85 cases, 19 (22.3%) were positive for CK5/6. Overall, 13 out of these 19 cases fell in the TNC category (68%, p = 0.001). No case from the Luminal A class showed expression for CK5/6 (0%, p = 0.028). Four cases of Luminal B class showed CK5/6 positivity (10.8%, p = 0.022) while two cases of HER-2-enriched class were positive for CK5/6 (33.3%, p > 0.05) (Table 1).

**Table 1:** Frequency of CK5/6 expression in different molecular subtypes. HER-2: human epidermal growth factor receptor 2

CK5/6 expression	Molecular subtype				Total
	Luminal A	Luminal B	HER-2 enriched	Triple-negative	
Negative	13	33	4	16	66
Positive	0	4	2	13	19
Total	13	37	6	29	85

Among the CK5/6-positive cases, the most common histological grade was grade 3 (G3) (57.8%, p = 0.20) and the pathological stage was pT3 (57.8%, p = 0.049).

LVI was present in eight of the CK5/6-positive cases (42%, p = 0.035). While none of the cases showed PNI (0%, p > 0.05). Regional lymph node metastasis was noted in 12 CK5/6-positive cases (63%, p > 0.05) (Table 2).

**Table 2:** Different prognostic parameters in relation to CK5/6-positive and negative expression.

Clinical parameters	CK5/6 expression		P-value
	Positive (n = 19)	Negative (n = 66)	
Lymphovascular invasion	8 (42%)	12 (18%)	0.035
Perineural invasion	0 (0%)	11(16%)	>0.05
Histological grade			
I	0 (0%)	0 (0%)	
II	8 (42%)	36 (54.5%)	>0.05
III	11 (57.8%)	30 (45%)	>0.05
Pathological stage			
pT1	0 (0%)	4 (0.06%)	>0.05
pT2	5 (25%)	32 (42%)	>0.05
pT3	11 (57.8%)	22 (33%)	0.049
pT4	4 (15.7%)	8 (12%)	>0.05
Regional lymph node status	12 (63%)	28 (42%)	>0.05

## Discussion

BC is one of the most common malignancies globally. The incidence of BC has increased by 123% between 1990 and 2017. The death toll of BC is high, with an estimated 1,503,694 deaths worldwide in 2025, suggesting that the global disease burden of BC will remain severe for some time to come [15].

BC comprises a heterogeneous group of tumors with variable clinical presentation, histological subtypes, biological behavior, prognosis, and treatment response [16]. The World Health Organization recognizes 18 different histological subtypes with IBC NST, previously known as IDC, being the most frequent type accounting for 40-80% of all BC cases. Other histological variants include invasive lobular carcinoma, mucinous carcinoma (types A and B), neuroendocrine, papillary, and salivary gland-type tumors [17-19].

**Figure 2:**
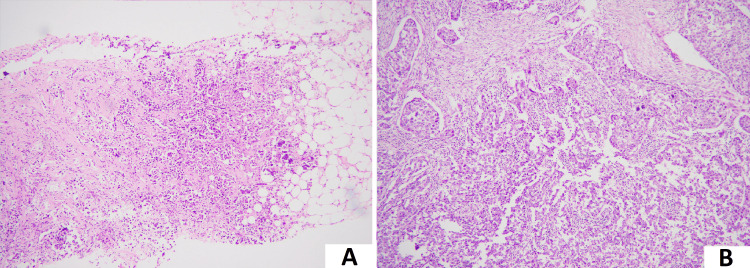
Histological features of invasive breast carcinoma. (A) Low-power image of invasive breast carcinoma of no special type, histological grade 2, demonstrating nests of tumor cells showing tubule formation and moderate pleomorphism (100× magnification). (B) Low-power image of invasive breast carcinoma of no special type, histological grade 3, demonstrating sheets of tumor cells showing marked nuclear pleomorphism (100× magnification).

The IBC NST is historically classified into different grades (G) with varying prognostic and predictive values. Prognostically, G1 is the best, while G3 is the worst. However, this grading is based on histological features only (as shown in Figure 2) and cannot accurately predict tumor biology and behavior.

Hence, these are further stratified based on mRNA gene expression in four main molecular subtypes, including Luminal A, Luminal B, HER-2-enriched, and TNC, which have better prognostic and predictive values [20,21]. TNC includes a spectrum of tumors with variable prognosis; however, the most common type within this class is grade 3 IBC NST which has poor response to conventional treatment regimens and overall prognosis [22-25]. Based on this variability, the triple-negative BC (TNBC) class has been extensively investigated for further histological or molecular signatures which may help in an improved understanding of tumor biology and help guide appropriate treatment options [26,27].

One of the most investigated areas in the TNBC class is the identification of tumors with basal-like phenotype which commonly expresses basal keratin CK5/6 [28-30]. Expression of CK5/6 has been widely investigated in other tumors, e.g., carcinomas of the urinary bladder and prostate, where it has conventionally been linked to poor prognosis [31-33]. For high-grade IBC NST, it is suggested in the literature to use CK5/6 as a surrogate marker to identify basal-like carcinomas and define poor prognosis [34,35].

**Figure 3:**
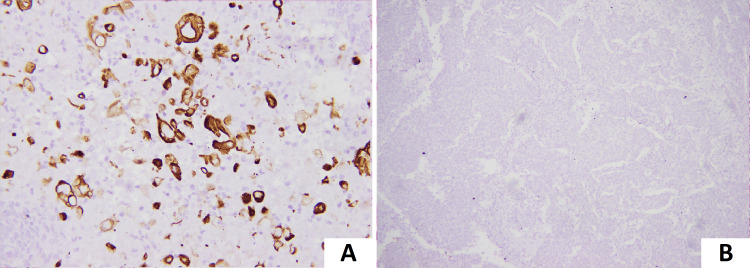
Expression of CK5/6. (A) Strong membranous expression of CK5/6 in tumor cells on immunohistochemistry. (B) Lack of membranous expression of CK5/6 in tumor cells on immunohistochemistry.

In this study, we evaluated the frequency of expression of CK5/6 in IBC NST cases within different molecular subtypes of BC. We also aimed to establish a correlation between adverse pathological features and the expression of CK5/6 (Figure 3).

In our study, we observed that CK5/6 expression was most frequent in TNBC, which is concordant with the findings of Kumar et al. and Khan et al. with CK5/6 expression in 85.7% and 40% of TNBC cases, respectively [36,37]. This finding is also concordant with Munirah et al. who reported CK5/6 expression in 100% of TNBC cases [38]. The Luminal B class had the second most common expression of CK 5/6 (10.8%) in our study which is concordant with Munirah et al. who reported it to be 7.1% while discordant with the findings of Kumar et al. who reported it to be 0% [36,38]. Luminal A showed no expression of CK5/6 in our study which is in concordance with the findings of Kumar et al. [36]. The HER-2-enriched class showed a 10% expression of CK5/6 which is concordant with the findings of Kumar et al. [36].

Expression of CK5/6 directly correlated with high histological grade (G3) which is concordant with the findings of Kumar et al. and Sowjanya et al. while discordant with the findings of Ara et al. [36,39,40].

Expression of CK5/6 showed a direct correlation with positive nodal status in our study. This finding is concordant with the findings of Sowjanya et al., Constantinou et al., and Tiwari et al., demonstrating 66%, 56.8%, and 91% of the cases, respectively, with CK5/6 expression and positive nodal status [40-42]. No correlation of CK5/6 expression was established with PNI in our study.

The majority of the cases with CK5/6 expression were high-stage tumors (pT3) in our study. The majority of cases with CK5/6 expression had a high pathological stage at the time of diagnosis. This is a significant finding that has not been adequately discussed in previous literature.

LVI was found in less than half of CK5/6-positive cases in our study which is concordant with the findings of Khan et al. [37]. However, Kumar et al. reported it to be in more than half of CK5/6-positive cases [36].

## Conclusions

The expression of CK5/6 in IDC of the breast is associated with poor prognostic factors such as the triple-negative molecular subtype, high histological grade, LVI, positive nodal status, and high pathological stage at the time of diagnosis.

## References

[1] Sung H, Ferlay J, Siegel RL, Laversanne M, Soerjomataram I, Jemal A, Bray F (2021). Global Cancer Statistics 2020: GLOBOCAN estimates of incidence and mortality worldwide for 36 cancers in 185 countries. CA Cancer J Clin.

[2] Lei S, Zheng R, Zhang S (2021). Global patterns of breast cancer incidence and mortality: a population-based cancer registry data analysis from 2000 to 2020. Cancer Commun (Lond).

[3] Siegel RL, Miller KD, Wagle NS, Jemal A (2023). Cancer statistics, 2023. CA Cancer J Clin.

[4] Zaheer S, Shah N, Maqbool SA, Soomro NM (2019). Estimates of past and future time trends in age-specific breast cancer incidence among women in Karachi, Pakistan: 2004-2025. BMC Public Health.

[5] Yin L, Duan JJ, Bian XW, Yu SC (2020). Triple-negative breast cancer molecular subtyping and treatment progress. Breast Cancer Res.

[6] do Nascimento RG, Otoni KM (2020). Histological and molecular classification of breast cancer: what do we know?. Mastology.

[7] Smolarz B, Nowak AZ, Romanowicz H (2022). Breast cancer-epidemiology, classification, pathogenesis and treatment (review of literature). Cancers (Basel).

[8] Al-Thoubaity FK (2020). Molecular classification of breast cancer: a retrospective cohort study. Ann Med Surg (Lond).

[9] Hon JD, Singh B, Sahin A (2016). Breast cancer molecular subtypes: from TNBC to QNBC. Am J Cancer Res.

[10] Kashyap D, Pal D, Sharma R (2022). Global increase in breast cancer incidence: risk factors and preventive measures. Biomed Res Int.

[11] Johnson KS, Conant EF, Soo MS (2021). Molecular subtypes of breast cancer: a review for breast radiologists. J Breast Imaging.

[12] Kayahan M, İdiz UO, Gucin Z, Erözgen F, Memmi N, Müslümanoğlu M (2014). Cinical significance of androgen receptor, CK-5/6, KI-67 and molecular subtypes in breast cancer. J Breast Health.

[13] Hashmi AA, Naz S, Hashmi SK (2018). Cytokeratin 5/6 and cytokeratin 8/18 expression in triple negative breast cancers: clinicopathologic significance in South-Asian population. BMC Res Notes.

[14] Alshareeda AT, Soria D, Garibaldi JM, Rakha E, Nolan C, Ellis IO, Green AR (2013). Characteristics of basal cytokeratin expression in breast cancer. Breast Cancer Res Treat.

[15] Xu Y, Gong M, Wang Y, Yang Y, Liu S, Zeng Q (2023). Global trends and forecasts of breast cancer incidence and deaths. Sci Data.

[16] Fitzmaurice C, Allen C, Barber RM (2017). Global, regional, and national cancer incidence, mortality, years of life lost, years lived with disability, and disability-adjusted life-years for 32 cancer groups, 1990 to 2015: a systematic analysis for the global burden of disease study. JAMA Oncol.

[17] Łukasiewicz S, Czeczelewski M, Forma A, Baj J, Sitarz R, Stanisławek A (2021). Breast cancer-epidemiology, risk factors, classification, prognostic markers, and current treatment strategies-an updated review. Cancers (Basel).

[18] Shiovitz S, Korde LA (2015). Genetics of breast cancer: a topic in evolution. Ann Oncol.

[19] Tan PH, Ellis I, Allison K (2020). The 2019 World Health Organization classification of tumours of the breast. Histopathology.

[20] Erber R, Hartmann A (2020). Histology of luminal breast cancer. Breast Care (Basel).

[21] Makki J (2015). Diversity of breast carcinoma: histological subtypes and clinical relevance. Clin Med Insights Pathol.

[22] Prat A, Cheang MC, Martín M (2013). Prognostic significance of progesterone receptor-positive tumor cells within immunohistochemically defined luminal A breast cancer. J Clin Oncol.

[23] Ades F, Zardavas D, Bozovic-Spasojevic I (2014). Luminal B breast cancer: molecular characterization, clinical management, and future perspectives. J Clin Oncol.

[24] Prat A, Carey LA, Adamo B (2014). Molecular features and survival outcomes of the intrinsic subtypes within HER2-positive breast cancer. J Natl Cancer Inst.

[25] Plasilova ML, Hayse B, Killelea BK, Horowitz NR, Chagpar AB, Lannin DR (2016). Features of triple-negative breast cancer: analysis of 38,813 cases from the national cancer database. Medicine (Baltimore).

[26] Santonja A, Sánchez-Muñoz A, Lluch A (2018). Triple negative breast cancer subtypes and pathologic complete response rate to neoadjuvant chemotherapy. Oncotarget.

[27] Pareja F, Geyer FC, Marchiò C, Burke KA, Weigelt B, Reis-Filho JS (2016). Triple-negative breast cancer: the importance of molecular and histologic subtyping, and recognition of low-grade variants. NPJ Breast Cancer.

[28] Badve S, Dabbs DJ, Schnitt SJ (2011). Basal-like and triple-negative breast cancers: a critical review with an emphasis on the implications for pathologists and oncologists. Mod Pathol.

[29] Wang DY, Jiang Z, Ben-David Y, Woodgett JR, Zacksenhaus E (2019). Molecular stratification within triple-negative breast cancer subtypes. Sci Rep.

[30] Shawarby MA, Al-Tamimi DM, Ahmed A (2013). Molecular classification of breast cancer: an overview with emphasis on ethnic variations and future perspectives. Saudi J Med Med Sci.

[31] Akhtar M, Rashid S, Gashir MB, Taha NM, Al Bozom I (2020). CK20 and CK5/6 immunohistochemical staining of urothelial neoplasms: a perspective. Adv Urol.

[32] Hashmi AA, Hussain ZF, Irfan M, Edhi MM, Kanwal S, Faridi N, Khan A (2018). Cytokeratin 5/6 expression in bladder cancer: association with clinicopathologic parameters and prognosis. BMC Res Notes.

[33] Lu JG, Lo ET, Williams C, Ma B, Sherrod AE, Xiao GQ (2023). Expression of high molecular weight cytokeratin-a novel feature of aggressive and innate hormone-refractory prostatic adenocarcinoma. Prostate.

[34] Schulmeyer CE, Fasching PA, Häberle L (2023). Expression of the immunohistochemical markers CK5, CD117, and EGFR in molecular subtypes of breast cancer correlated with prognosis. Diagnostics (Basel).

[35] Nielsen TO, Hsu FD, Jensen K (2004). Immunohistochemical and clinical characterization of the basal-like subtype of invasive breast carcinoma. Clin Cancer Res.

[36] Kumar N, Patni P, Agarwal A, Khan MA, Parashar N (2015). Prevalence of molecular subtypes of invasive breast cancer: a retrospective study. Med J Armed Forces India.

[37] Khan SP, Yasin SB, Khan FP (2017). A comparative study of clinicopathological characteristics and expression of basal markers (CK5/6 & EGFR) in triple negative and non triple negative breast carcinomas in Kashmir Valley. Ann Appl Bio-Sci.

[38] Munirah MA, Siti-Aishah MA, Reena MZ (2011). Identification of different subtypes of breast cancer using tissue microarray. Rom J Morphol Embryol.

[39] Ara NJ (2021). Determining the expressions of cytokeratin 5/6 by immunohistochemistry in basal like triple negative breast carcinoma and its correlation with histomorphological grade. SAS J Med.

[40] Sowjanya R, Divyagna T, Goud SD, Jyothi C, Swapna B, Kumari KY (2023). Expression of cytokeratin 5/6 in benign and malignant breast lesions. Int J Acad Med Pharm.

[41] Constantinou C, Papadopoulos S, Karyda E, Alexopoulos A, Agnanti N, Batistatou A, Harisis H (2018). Expression and clinical significance of Claudin-7, PDL-1, PTEN, c-Kit, c-Met, c-Myc, ALK, CK5/6, CK17, p53, EGFR, Ki67, p63 in triple-negative breast cancer-a single centre prospective observational study. In Vivo.

[42] Tiwari S, Malik R, Trichal VK (2015). Breast cancer: correlation of molecular classification with clinicohistopathology. Sch J App Med Sci.

